# Why is it so hard to reach agreement on terminology? The case of developmental language disorder (DLD)

**DOI:** 10.1111/1460-6984.12335

**Published:** 2017-07-17

**Authors:** Dorothy V. M. Bishop

**Affiliations:** ^1^ University of Oxford Oxford UK

**Keywords:** Developmental language disorder, Consensus, Delphi, CATALISE, Labels, Diagnosis

## Abstract

A recent project entitled CATALISE used the Delphi method to reach a consensus on terminology for unexplained language problems in children. ‘Developmental language disorder’ (DLD) was the term agreed by a panel of 57 experts. Here I reflect on points of difficulty that arose when attempting to reach a consensus, using qualitative information from comments made by panel members to illustrate the kinds of argument used. One issue of debate was the use of labels, in particular the term ‘disorder’, which was seen as having both pros and cons. The potential for labels to stigmatize or create low expectations was a particular concern. However, labels could also ensure language problems were not trivialized and could help avoid stigma by providing an explanation for behaviours that might otherwise meet with disapproval. Further debate surrounded issues of how best to identify cases of disorder. Although it was agreed there should be a focus on cases with a poor prognosis, it was recognized that our knowledge of factors related to prognosis was still incomplete. Furthermore, there was a tension between use of standardized tests, which allow for a relatively objective and reliable assessment of language, and more qualitative observations, which can capture functional aspects of communication that are not always picked up on formal assessment. Debate also surrounded the issue of the relationship between DLD and other conditions. Some favoured drawing a distinction between DLD and language disorders associated with other conditions, and others regarded such distinctions as unnecessary. We concluded that it was misleading to assume co‐occurring conditions were causes of language disorder, but it was helpful to distinguish DLD from cases of language disorder associated with ‘differentiating conditions’ that had a known or likely biomedical origin, including brain injury, sensorineural hearing loss, genetic syndromes, intellectual disability and autism spectrum disorder. Furthermore, DLD could co‐occur with milder neurodevelopmental disorders that did not have a clear biomedical aetiology. Normal‐range non‐verbal IQ has traditionally been incorporated in the diagnosis of DLD, but this was rejected as unsupported by evidence. DLD is a category that has utility in identifying children who would benefit from speech–language therapy services, but it should not be thought of as a well‐defined condition. DLD has a multifactorial aetiology, is heterogeneous in terms of language features and overlaps with other neurodevelopmental disorders. Our notions of DLD are likely to be refined by further research into aetiology, associated characteristics and intervention effectiveness.

**What this paper adds**

**What is already known on the subject**

Children's language disorders have received far less recognition than other neurodevelopmental conditions such as attention deficit hyperactivity disorder (ADHD), autism spectrum disorder and developmental dyslexia. In part this is a result of inconsistent terminology and definitions of language disorder. As a consequence, there is disagreement as to which children should receive intervention.

**What this paper adds to existing knowledge**

In 2016–17, two linked projects were carried out using the Delphi method to work towards a consensus in the criteria and terminology used for children's language disorders. In this paper I use qualitative comments from the evaluation panel to explore the reasons for debate in this area. Although some disagreements reflect an inadequate research base, other issues involve fundamental questions about the impact and validity of diagnostic labels, and the criteria for deciding who receives intervention.

**What are the potential or actual clinical implications of this work?**

Clinicians who wish to adopt the new terminology from the CATALISE study may find these reflections helpful in clarifying the clinical and theoretical reasons behind the consensus statements. The paper also emphasizes that the category of ‘developmental language disorder’ (DLD) is a useful shorthand rather than a specific syndrome—a hypothesis to be refined in the light of new discoveries.

In 2015–16, an international group of experts, the CATALISE panel, came together with the aim of achieving consensus on diagnostic terminology for children's language disorders. Ebbels (2014) documented the history of this development, with the need for consensus being emphasized by several converging forces. First, there was mounting evidence that children's language disorders received scant research funding in relation to their frequency and severity (Bishop 2010). Then in 2012, a live debate entitled ‘What IS Specific Language Impairment’ took place at Moor House School, a specialist school for children and young people with speech and language difficulties. This failed to reach agreement about how to diagnose or define specific language impairment (SLI). In addition, a group that had started a campaign for Raising Awareness of Language Learning Impairments (RALLI) (Bishop *et al*. 2012) was confronted with the difficulty of raising awareness of a condition that was ill‐defined, and where terminology was inconsistent. In 2013, Sheena Reilly, in the *IJLCD* Winter Lecture raised numerous questions about the usefulness and validity of the term ‘SLI’, using data from an Australian epidemiological study to argue that the ‘specific’ part of the label was counterproductive and misleading—views that were subsequently articulated in this journal (Reilly *et al*. 2014b). To complement that paper, I wrote a review outlining the major issues where consensus was lacking, and suggesting various solutions (Bishop 2014). Both papers appeared in a special issue of this journal (Ebbels 2014), accompanied by commentaries from experts covering a range of professional groups and interests. The authors of the original focus papers then collaborated to highlight the main points of agreement and disagreement in the debate (Reilly *et al*. 2014a).

Starting in 2015, a two‐stage project, CATALISE (Criteria and Terminology Applied to Language Impairments: Synthesising the Evidence), was developed with the aim of yielding consensus statements about children's language disorders (Bishop *et al*. 2016, 2017). The method adopted was the Delphi Survey Technique (Hasson *et al*. 2000). This involves working with a panel of experts who are provided with a set of statements that they both rate and comment on. Panel members were encouraged to cite research evidence to justify their viewpoints. The process is iterative, with a new set of statements being formulated on the basis of the previous round. This exercise, when conducted online, has several advantages over in‐person discussion. For a start, panel members have time to think about the issues and to complete their ratings at a time that is convenient. This makes it possible for people to take part from all over the world.^1^ Furthermore, each person's ratings and comments are fed back anonymously to the whole panel. Anonymity avoids the discussion being dominated or derailed by a few forceful individuals. If the feedback indicates that one's point of view is unpopular, it is possible to present arguments and evidence to try to persuade others, or to revise one's ratings. Two moderators (Dorothy Bishop and Maggie Snowling), who were not part of the panel, integrated the information from each round, using the comments accompanying the ratings to identify the reasons for disagreement and to revise statements to address these. The first Delphi exercise focused on criteria and the second on terminology.

One outcome of this exercise was the recommendation that the term ‘specific language impairment’ (SLI) be abandoned in favour of ‘developmental language disorder’ (DLD). Rather than recapitulating the conclusions of the two Delphi exercises, which are covered in depth in two papers (Bishop *et al*. 2016, 2017), my aim here is to use the comments offered by panel members during these projects to reflect on the factors leading to change and in particular to note reasons for controversy in this area.

However, it is worth first emphasizing issues on which there was good agreement from the outset. All panel members recognized that some children have difficulties mastering their native language, and that these can be severe enough to impair their ability to function well in educational and social contexts. In many cases there is no obvious cause. A second point was that it was important to reach agreement, because lack of consensus had been damaging to the field, and did a disservice to affected children and their families.

It was also agreed that language disorders in children can take many forms. The classic ‘textbook’ case is of a child whose language level is well below that of their peers—for instance, a 4‐year‐old who talks more like a typical 2‐year‐old, with simplified grammar and limited vocabulary. Where the problems are as striking as this, language difficulties are not hard to recognize, but what of the child who has more subtle problems, or where the difficulties involve the use rather than the form of language? A core notion is that language is out of keeping with the child's general developmental level, but any attempt to turn this idea into more specific criteria is fraught with difficulties, not least because language is a complex, multidimensional skill, which changes rapidly with age.

We had expected that where there were disagreements about definitions and terminology these might divide along professional or national lines, but, as far as our analysis could establish, this was not the case. Rather, it seemed that where differences of opinion existed, it was because of lack of evidence, or else because all solutions that were discussed had disadvantages as well as advantages. In effect, there was no gold standard against which to evaluate terminology, and no ideal solution. This creates an unstable situation because whatever solution is adopted, problems will be noted, and there will therefore be pressure for further change.

It may seem counterproductive to highlight points of disagreement concerning the consensus statements that we arrived at. If we want people to endorse the new terminology, would it not be better to draw a veil over the more contentious aspects? In my opinion, this would reduce the credibility of the exercise by suggesting difficult issues were not grappled with. Furthermore, only by being aware of both pros and cons can we mitigate any negative consequences of new criteria and terminology, while promoting the benefits.

## Why do we need categorical labels?

Before we address the question of which labels to use, a more primary question is whether it makes sense to use labels at all. Most panel members took the need for an agreed label as a given:

I think this is one of the reasons that children's language difficulties are not taken as seriously or recognized in the same way as other conditions such as dyslexia or ASD [autism spectrum disorder]. There are too many different terms, that are being used in too many different ways, so people are just not really paying attention any more.

This is important across the board—for epidemiological purposes, for clarity in individual research studies, and for policy makers and service providers.

Given the lack of awareness of language disorder in both professional and public fields, it is really important that we as professionals with knowledge of this area can reach a consensus on the terminology so that we can move forward with providing services and recognition for these children and their families.

However, labels influence how we think about constructs, with words dividing the world into distinct entities such as cats, dogs, desks and tables. Yet, in practice, some of these entities are not clearly demarcated: the boundary between desk and table, for instance, is not sharp. Diagnostic terms can create an insidious problem whereby the label is taken to mean that the child has a clear‐cut disorder with distinctive ‘symptoms’, which can be distinguished from both typical development and other developmental disorders. In fact, there is no support for a sharp dividing line between language disorder and normality. Children develop language at different rates, and some will have problems severe enough to cause lifelong problems. But the differences between children appear to be quantitative rather than qualitative, and can take many different forms (Leonard 1991, Dollaghan 2011). Much research literature has focused on limitations of expressive syntax in children with DLD, but as our assessments become more fine‐grained, it is evident that problems often affect understanding as well as production of complex language, and can involve phonology, semantics and pragmatics as well as grammatical structure.

It is all too easy for a label to become a master rather than a servant, if we over‐interpret its significance, rather than regarding it as a useful shorthand. It is therefore important that if we use labels, we do so with full awareness of their limitations, and recognizing that our terms are socially constructed and historically specific. The term we agreed on, ‘DLD’, is defined in terms of behaviours that are complex, multifactorial and which vary on a continuum. It is an umbrella term that includes a wide range of problems, and the boundaries between DLD and typical development are fuzzy. One panel member noted that in the field of educational psychology some people were turning away from the label ‘developmental dyslexia’ for children with reading problems precisely because of arguments such as these (Elliott and Grigorenko 2014).

Another panel member noted a further problem caused by labels: their potential to stigmatize children:

I am increasingly concerned that the exercise risks generating labels/categories for children that will be operationalized to allocate resources and will discriminate against positive expectations for more than is scientifically justified.

Given these risks of misinterpretation, we need to consider whether we could do away with labels altogether. It was concluded, however, that this would create more problems than it solved. As I have discussed previously (Bishop 2014), diagnostic labels have utility in clinical as well as research contexts. Labels allow us to communicate efficiently in a common language: without labels, it would be difficult to plan services and to decide how to allocate them. This issue is not specific to DLD: it pervades all of psychiatry, including child psychiatry. As Sonuga‐Barke (1998: 117) noted, categorization is a practical necessity in clinical practice:

To the extent that clinical economy depends on getting the right treatment to the right people, clinicians are, no matter what their philosophical bent or political point of view, categorisers. At a purely practical level this depends on a judgement being made that such and such a child belongs to the category of those who ‘need help’, whereas another child belongs to a (usually) larger category who do not.

Nonetheless, at the same time as we strive to get better resources to help those with DLD overcome their problems, we need to consider how best to create conditions to combat stigma. A first point to note is that research on children with DLD suggests that stigmatization is often a reaction to the child's communication difficulties (Macharey and von Suchodoletz 2008). And experience with other conditions suggests that labels can actually help reduce stigma by improving understanding. In the field of autism, for instance, a recent study found that university students were more accepting of socially atypical behaviour when they were told that the person had a clinical diagnosis (of Asperger syndrome, autism spectrum disorder (ASD) or schizophrenia) (Brosnan and Mills 2016). *One study found that while a general label of ‘special educational needs’ was associated with low self‐esteem, a more specific label* (in the case of this study, ‘dyslexia’) was not (Taylor *et al*. 2010). In the field of mental health, there is some evidence that self‐identifying with a stigmatized group can buffer the individual against some of the negative effects (Crabtree *et al*. 2010). Such research suggests that in raising awareness of terminology, we should aim to provide information in a way that will lead to better acceptance and willingness of others to make adjustments to take into account language limitations, and to facilitate individuals with DLD coming together for mutual support. Second, we need to communicate diagnosis to affected individuals and their families in a way that emphasizes that DLD does not preclude achieving well in non‐linguistic domains and being a well‐integrated member of society. We need to celebrate the achievements of those who have succeeded despite their language difficulties. It is worth noting that I have searched the internet for examples of positive role models of people with DLD and not been able to find any—in sharp contrast to dyslexia and ASD, where such role models are numerous.

## Use of the term ‘disorder’

Most of the panel welcomed the move towards a single agreed term to refer to children with persistent language problems, but there was much debate about the use of the term ‘disorder’. As discussed by Bishop (2014), in the field of education other terms such as ‘difficulties’ and ‘needs’ have been used. Other options are ‘impairment’ and ‘disability’. These are sometimes used interchangeably, and sometimes with subtly different meanings. ‘Disorder’ was preferred by the CATALISE panel for two reasons. First, it is the term used in DSM5 and ICD‐11, both for language problems and for other neurodevelopmental conditions, especially ASD, attention deficit disorder, and developmental coordination disorder. It was deemed counterproductive to use terminology that was at odds with those widely used systems. Second, as one panel member said:

I feel it communicates the seriousness of the issue more effectively than ‘impairment’.

A handful of panel members felt ‘disorder’ did not go far enough, and that a more medical‐sounding label would be more effective:

We need a label with some authority. Once again, I really do have to go back to the suggestion of dysphasia, on analogy with dyslexia and dyspraxia. Terms like this have the advantage of sounding like real conditions (which is why parents will fight so hard for a ‘diagnosis’ of dyslexia). People sit up and take notice of it.

However, among commentators in the special issue, the label ‘dysphasia’ had been one of the most unpopular options. This illustrates a dilemma for the field: the labels that sound most ‘medical’ are seen as misleading by professionals, yet they are the terms that are most likely to attract sympathy and attention. The parallel with dyslexia is very moot here: although dyslexia is not a coherent syndrome (Elliott and Grigorenko 2014), moves to drop the term from DSM5 were strongly resisted, with the International Dyslexia Association mounting a petition for its reinstatement, citing concerns that diagnosis and intervention would be delayed if the more generic and neutral term ‘specific learning disorder’ were used instead (International Dyslexia Association 2012).

At the other extreme, there were concerns again about reification of the term DLD, and stigmatization and negative connotations.

The use of ‘disorder’ will be perceived as denoting a state of being essentially, categorically, different and distinct from those on the ‘normal’ distribution.

Although I agree with the term and the explanation given, the use of the word ‘disorder’ has very negative connotations for teachers and those within educational policy. … There is no useful term that gets around this issue, however.

The different viewpoints illustrated by these quotes emphasize the difficulty of finding an ideal solution. If we use a term that reflects the fact that children have serious problems and need support, this comes across as negative and could create a self‐fulfilling prophecy; however, if we use milder language that is not deemed stigmatizing, then we run the risk that children's problems may be trivialized, with consequent delays in identification and intervention.

### Traditional distinction between ‘disorder’ and ‘delay’

A quite different objection to the term DLD is that, at least in the UK and some parts of the Irish Republic, ‘disorder’ has been interpreted contrastively as a condition different from ‘language delay’. Indeed, we learned that many speech–language therapists (SLTs) in the UK had been trained to make this distinction. It embodies the idea that there is a particular profile of language difficulties, where there is a large mismatch between non‐verbal ability and language skills, and also an uneven profile of ability within the language domain. This profile is seen as the hallmark of ‘disorder’, whereas ‘delay’ is thought to be characterized by an even pattern of impairment, so that both verbal and non‐verbal skills are like that of a younger child. This distinction has assumed considerable importance in some regions, where SLT services are allocated only to children with ‘disorder’, and not to those with ‘delay’—presumably because ‘delay’ is interpreted literally as indicating that the child will catch up after a slow start.

It is hard to trace the origins of this distinction, but it is clear that, despite its superficial plausibility, it does not have empirical support. Thirty years ago, Bishop and Edmundson (1987) used longitudinal data to test the idea that an even pattern of language impairment had a better prognosis than a more ‘spikey’ profile, with selective weakness in some domains. Their results were opposite to prediction: those with more selective problems had the best outcomes, whereas those with more general across‐the‐board impairments had the worse prognosis. Thus, providers who restrict SLT services from those who have ‘delayed’ profiles are denying help to those children who are most in need. Nor is there any evidence that speech–language therapy is only effective for children with a large mismatch between language and non‐verbal skills (Cole *et al*. 1990), which is why the American Speech–Language–Hearing Association does not support ‘cognitive referencing’, i.e., using the discrepancy between non‐verbal IQ and language as a basis for allocating services (American Speech–Language–Hearing Association 2016). Given the widespread use of the delay/disorder distinction, it is vital that those using DLD are clear that, as used by the CATALISE panel, the term ‘disorder’ does not entail any discrepancies, either between verbal and non‐verbal development, or between different language functions.

## Criteria for language disorder: focus on poor prognosis

The definition of DLD agreed by the panel applies specifically to children with poor prognosis. This averted a concern that children with mild and transient difficulties might be labelled as ‘disordered’. However, a focus on poor prognosis does raise concerns that children might be written off, or that parents will be discouraged if they are told that their child's problems are unlikely to resolve with time. Most panel members agreed that this criterion made sense, and indeed could lead to fairer allocation of resources, but it is clear that it needs to be applied cautiously:

I think the focus on prognosis is important. It also makes clear that the children who require intervention are the children with a poor prognosis. Currently in many areas of the UK children with poor prognosis are not receiving services because they clog up the system and can't be discharged easily! The focus on the tier 3 children who won't make progress without help may help to counter this shift in service delivery.^2^

There were, however, two concerns: first, did we know enough about prognostic factors to be able to judge this appropriately?; and second, would this mean denying services to children who would benefit from intervention?

we need to look further into the research and agree more solid indicators of long‐term language learning difficulties.

There was variable awareness among CATALISE members of existing research on predictors of outcome. Our panel included several experts who had been involved in running longitudinal studies and were in general supportive of using prognosis as a criterion, but others had concerns about the feasibility of identifying children who were likely to have longstanding problems. Between these extremes were those who thought that even if we have good predictors from research, they may apply only to the specific population that was studied, and prediction may also not be precise enough to make accurate judgements about individual children. Over and above this concern, was the question of whether children with good prognosis should be excluded from specialist help.

I think it is important not to exclude children who may have, for example, very poor expressive language, which is causing them considerable difficulties, but might respond very well to specialist input. … Even if they do catch up reasonably quickly in relative terms, the children might still have lost ground and had a miserable couple of years, which might itself have long lasting effects.

This latter point emphasized that the criteria adopted could change the balance of which children would gain access to resources. Throughout the process, comments were made which indicated how seriously panel members took this: many thought that current allocation of SLT services was not fair, but others worried that a move to a different set of criteria might leave children who might benefit from SLT intervention without any help. The impression was that, for those with clinical caseloads, views were strongly influenced by personal experience: it was clear that most panel members were concerned that large numbers of children were unfairly excluded from access to services, but while some maintained that those with the most severe problems were denied help, others took the opposite view and noted that it was children with milder and more selective language difficulties who risked being excluded from intervention.

This debate emphasized the lack of a good evidence base for making rational decisions in this area. Of course, we do not want to expend scarce resources on children who will either improve without help, or who are unlikely to benefit from intervention. But in the absence of a strong evidence base, it seemed that subjective impressions were often used to distinguish these groups. One point where the evidence was clear concerned the difficulty of predicting outcomes in late‐talking toddlers: a diagnosis of DLD would be difficult to justify in a child under 3 years of age unless there were notable comprehension difficulties. In cases where the prognosis is hard to judge, which would include many pre‐school children, the more generic term ‘speech, language and communication needs’ (SLCN), already in widespread use in the UK, would seem more appropriate for flagging up problems than the more clinical diagnosis of DLD.

As reviewed by Bishop *et al*. (2017), we do have better prognostic indicators for children from around 5 years of age when it becomes reasonable to identify children whose problems are unlikely to resolve without help. Nevertheless, it is frustrating that even when we have evidence from longitudinal studies, the clinical application of the findings is often limited because of an emphasis on demonstrating that a predictor is statistically significant, rather than on its effectiveness in predicting individual outcomes.

Also, there can be difficult decisions even when evidence is available: should resources go to those who can easily be treated effectively, or to those with the most severe functional impairments? These are unlikely to be the same children.

In addition, this raises the question of what the goals of intervention should be. It might seem obvious that a successful intervention is one that improves the child's language skills so that they are at the level of the peer group. Yet for some children, more modest goals—of providing strategies to cope with language difficulties, and modifying the environment to make the language impairments less detrimental—may be more realistic. If evaluation studies focus solely on language tests as outcome measures, then this may underestimate the benefits of intervention. To get a more realistic impression of outcomes, measures of quality of life, family functioning, social integration and self‐esteem should be included.

Overall, it became clear that a discussion that was on the surface about diagnostic criteria was heavily influenced by beliefs about which groups should be given priority when intervention resources were scarce.

### Criteria for language disorder: How objective and standardized should we be?

Further tensions were revealed between those who felt it was important to use objective measures with strong psychometric credentials and to avoid reliance on subjective judgements, and those who thought that formal language assessments failed to capture key features of children's language problems and might both over‐ and under‐diagnose problems.

The first quotation illustrates the former concern, the second the latter:

I have concerns about relying on parents/teacher identification. Parents may not have enough information about typical language development and/or may miss comprehension difficulties. Also, there are equity issues. Children whose caregivers are least able to identify language issues may be children particularly in need of support.

I agree that testing is important, however I feel that in order to establish severity, functional assessment (informal observation for example) must be included as well as standardized testing.

Concerns were also expressed about poor sensitivity and specificity of standardized language assessment batteries, and their inability to detect some important aspects of impairment, such as pragmatic difficulties:

single tests measuring single components of the language system often do not possess the most robust psychometric properties.

we need to discuss the tools that are used to identify these problems as they have many flaws and it is likely that a multiple of assessment types, different for each age and skill level will be needed.

The consensus of the CATALISE panel was that test scores provided useful information but should not be used as the sole criterion for identifying language disorder. It was key that there should be evidence of functional impairment, i.e., the language problem impacted on the child's social interaction and/or educational progress. Nevertheless, it was noted that we lack good assessments for many aspects of language, and that reliance on subjective judgements created scope for biased and inequitable decisions.

## Differentiating conditions rather than exclusionary criteria

Exclusionary criteria have traditionally been a key part of the diagnosis of SLI, but this part of the definition has been roundly criticized, notably by Reilly *et al*. (2014b). The CATALISE panel had many deliberations over whether we should differentiate between children whose language difficulties had no obvious cause and those whose difficulties occurred in the context of a specific aetiology, such as brain damage, hearing loss or a genetic syndrome. We concluded that where there is a differentiating condition ‘X’, the term ‘language disorder with X’ would be used rather than DLD. This distinction is important in many research contexts, where it may be desirable to focus on a relatively homogeneous group.

The discussion revealed several different factors that led to tension. Implicit in the debate are three issues. First, can we regard a co‐existing condition as the cause of the language disorder? Second, does the presence of a specific aetiology mean that no intervention should be offered for language disorder? Third, if intervention is offered, should it be of a different kind than for children with no obvious aetiology? In addition, the answers to all these questions will depend on exactly what we regard as a co‐existing condition.

The first issue may be illustrated with the case of hearing loss. A severe or profound congenital hearing loss is a major risk factor for poor development of oral language. However, if the child has exposure to signed language, good language skills are usually demonstrated. Similarly, many children can make good progress in oral language if given a cochlear implant. Nevertheless, there are hearing‐impaired children who have problems learning sign language, and others who have disproportionate difficulties with oral language acquisition after a cochlear implant (Hawker *et al*. 2008). And this should not surprise us: we should expect to see a proportion of children with hearing loss who also have genetic risk factors for language disorder. While this example illustrates the dangers of explaining away a child's language disorder in terms of an associated condition, it is not an argument for simply ignoring the associated condition when identifying language disorder. It is rather an argument for documenting carefully the range as well as the nature of language problems associated with a specific aetiology.

The discussion about causality was pervaded by concerns that if a child's language disorder was thought to be ‘explained’ by an associated condition, then no intervention would be offered. Although this has sometimes been used as a justification for denying services to children, it is not a logically coherent stance. If extended to medicine as a whole, it would mean that we would only offer treatment for conditions of unknown aetiology! The panel agreed that it is not appropriate to deny intervention to a child because they has a co‐existing condition, provided the child meets criteria for language disorder (in terms of severity and prognosis).

[The term ‘language disorder associated with X’] is a great idea not only because it explicitly describes the association, but also because it sends the important message that problems in one area do not ‘protect’ children from having other difficulties. Parents of children with associated difficulties (e.g. Down syndrome) are sometimes frustrated by the lack of recognition of their child having a language disorder on top of the language difficulties associated with Down syndrome. And of course there is the important work on ASD with and without language disorder.

On the third issue, many felt that a different approach to intervention might be required if the child's language problems occurred in the context of a known aetiology. To continue with the example of hearing loss, intervention might include giving the child a hearing aid or cochlear implant, and/or introducing sign language, or having an SLT focus on presenting information via the visual modality. It was noted that there were many disorders where it helped to make this distinction:

I do agree that there are children who have disordered language for various reasons who would not best fit into the traditional ‘SLI’ care pathways.

There was, however, a contrasting view that children with differentiating conditions might not be offered interventions which could be useful—just because it was assumed they would not work:

some children with ASD appear to have a primary grammatical language impairment which would not be typical of ASD, yet they are not offered the same intervention that other children showing these patterns of language learning would be offered.

it is important to note that they may NOT indicate a different treatment pathway. For example, milieu communication training has been shown to efficacious for children with language disorders associated both with ID and ASD.

I think our intervention research has been mostly directed to fairly narrowly defined categories, with some exceptions. So the presumed different intervention pathways might turn out to have more in common than we think—for example, similar approaches might affect significant change in the morpho‐syntactic difficulties of a child with Down syndrome and morpho‐syntactic difficulties in SLI.

The discussions of these issues made it clear that, once again, one reason for disagreements was lack of a good evidence base: very little was known about efficacy of language interventions for children with co‐existing biomedical conditions. The need for more research explicitly comparing different conditions was highlighted by our study. The best way to counteract concerns that budget‐holders would deny services to children with additional conditions would be to provide evidence that intervention is effective with these groups.

These arguments, together with the need for researchers to study relatively homogeneous groups, led to general agreement that we should distinguish between language disorder associated with a ‘differentiating condition’ and DLD, while recommending that in both cases the language disorder merited assessment and intervention.

The question, then, was what to include in ‘differentiating conditions’. We settled on a definition that included biomedical conditions such as a genetic syndrome, a sensorineural hearing loss or neurological disease. The conditions of ASD and intellectual disability led to some debate, but it was decided to include these as differentiating conditions, because, on the one hand, impairments of communication are a reliable feature of these conditions and, on the other, accelerating genetic advances mean that a biomedical aetiology is becoming evident for a high proportion of affected children (McRae *et al*. 2017). In practice, a differentiating condition would typically be the main diagnosis, with language disorder seen as a component of the condition.

## DLD in relation to other neurodevelopmental disorders

The notion of ‘differentiating condition’ does not encompass milder neurodevelopmental disorders of unknown origin, such as ADHD or developmental coordination disorder. These do not have a clear‐cut aetiology, and their association with language disorder is weaker than for the differentiating conditions noted above. One reason for rejecting the previous term ‘SLI’ was because ‘specific’ could be taken to imply that the child had no difficulties except with language. In contrast, our definition of DLD emphasizes that these idiopathic neurodevelopmental disorders can co‐occur with DLD. Because different professional groups are concerned with different conditions—educators with dyslexia, SLTs with DLD, paediatricians and psychiatrists with ADHD and ASD–children often receive a rather one‐sided assessment, depending on the professional they see. We emphasized that the fact that a child may have ADHD or dyslexia does not preclude a diagnosis of DLD and does not mean that the SLT should not be involved. The ways in which children's problems manifest does not neatly divide up according to professional specialities.

This view of overlap between neurodevelopmental disorders is supported by research on aetiology, which has shown that the causes of these conditions are often complex and multifactorial, resulting from the combined impact of many small genetic and environmental influences: the precise profile of problems that is seen in a child may depend on the particular constellation of aetiological factors, so we do not see sharp boundaries between conditions, but rather different permutations and combinations of impairments. Furthermore, although different disorders co‐occur at above chance levels, they can be dissociated, suggesting that they may be separate consequences of common risk factors.

Comments from panel members made it clear that the question of how to handle co‐occurring disorders was easier if one avoided attempting to specify causal relationships, e.g., if a child has both attentional problems and language problems, it is useful to be aware of this, but it may be impossible to disentangle whether attentional problems cause language problems or vice versa:

co‐occurring disorders could be defined as impairments in other cognitive or behavioural domains that can co‐occur with language disorder and may affect prognosis, but which do not include impairments of communication as core symptoms. I would avoid discussion of cause.

## The role of non‐verbal IQ in diagnosis

As discussed by Bishop (2014) and Reilly *et al*. (2014), traditionally, the diagnosis of SLI has required a non‐verbal IQ within normal limits. In some diagnostic systems, an even more stringent requirement of a large mismatch between verbal and non‐verbal ability is required. The CATALISE panel considered the evidence for this and concluded this criterion was not valid. Many panel members were supportive of dropping it.

I agree in so far as I do not want any discrimination on the basis of (non‐verbal or any other concept of cognitive) ability.

This is an important equity issue.

Again, the argument was that the focus should be on the child's language needs, rather than on putative aetiology. Others, however, noted this would be a major departure from current practice and expressed reservations about expanding the category of DLD, thereby placing pressure on services. Concern was expressed that a less restrictive definition of language disorder could lead to a watering down of the concept that would ultimately lead to a reduction in services:

If we lose the conceptualisation of language impairment as being a weakness in language skills relative to the child's average non‐verbal ability, then service providers might take the view that if there is no distinction between children of low ability across the board (who do not usually access a high level of therapy) and children with (specific) language difficulties, then it is not necessary to provide enhanced levels of SLT to any of them.

There also was evidence of local differences in how intervention resources were allocated. As noted above, practitioners from the UK and Republic of Ireland noted that a selective language problem (with normal non‐verbal ability) was usually required in order for the child to be offered SLT services, but in other places quite the opposite was described, and there was concern that children with more selective problems might lose out:

I regularly encounter situations where services have been denied to families because their children only presented with language impairments. The risk of under‐treatment in these cases is also what our epidemiological data have been telling us.

Overall, the view of the panel was that resources should not dictate diagnostic criteria. Discrepancy between verbal and non‐verbal ability is not indicative of underlying aetiology—for example, Bishop (1994) found that identical twins with language problems often had very similar language profiles, yet could vary in non‐verbal ability, with one meeting traditional discrepancy criteria for SLI, and the other not. Furthermore, for a child without a biomedical syndrome, the level of non‐verbal IQ does not appear to determine responsiveness to therapy. There does not, therefore, appear to be any justification for using a verbal–non‐verbal discrepancy as a diagnostic criterion; the overall view of the panel was that if this change led to more children being identified with DLD, then the case should be made for adequate resources to offer services to all of them.

Note that this does not mean that intellectual level is totally disregarded. As noted above, where the child meets criteria for intellectual disability, then we would treat that as the primary diagnosis and talk of ‘language disorder with intellectual disability’. The current DSM‐5 definition of intellectual disability requires that the child shows both ‘intellectual deficits and adaptive deficits that fail to meet the standards for personal independence’ (Harris 2013, 261).

Our definition of DLD would, however, include a case where a child with language problems has a level of non‐verbal ability that is neither impaired enough to justify a diagnosis of intellectual disability nor good enough to be discrepant with overall language level. These children have historically ‘fallen through the cracks’ of diagnostic systems, and hence been deemed ineligible for speech and language therapy services.

## Conclusions

Many of the issues raised by this analysis apply not just to language disorders but to neurodevelopmental disorders more broadly. Indeed, questions about diagnosis and terminology pervade all of psychiatry (Sonuga‐Barke 1998).

As we proceeded through the Delphi studies, we were confronted by a key question: How would we know when we had a good solution? In practice, in a Delphi exercise the goal is to achieve a reasonable agreement between experts, and we were satisfied to accept a solution where we had a set of statements that at least 75% agreed with. But, as is all too apparent in many walks of life, the fact that a lot of people agree with something does not make it true. Ideally we would like to be able to show that our solution worked better than what had gone before. But what does ‘work better’ mean?

The ideal would be to find a solution that ‘carves nature at its joints’, such that DLD would identify a group of children with a distinct aetiology, correlated features and response to intervention. However, as Sonuga‐Barke noted, this may be an unrealistic ambition if the reality is more dimensional than categorical. In the case of DLD, we have heterogeneity of language problems, and overlap between disorders affecting language and other domains. Despite this heterogeneity we have made some progress: our knowledge of aetiology, characteristics and effective intervention in this area has advanced over the years. Nevertheless, to improve our classification we need more research that carefully documents the nature of children's language difficulties in relation to prognosis, associated factors and response to intervention. The validity of the term ‘DLD’ will depend on whether it helps us move forward to a better understanding of what is clearly a heterogeneous category, bearing in mind that, as Sonuga‐Barke noted, the category is a hypothesis to be tested and refined. Meanwhile, it is to be hoped that a clearer specification of DLD and agreement about use of this term will lead to a more equitable and effective allocation of services to children who experience severe and persistent language problems.

## Notes

1 In CATALISE, we restricted the panel to participants from English‐speaking countries; it is clearly important to extend to other languages, but we were aware that the debate would be complex enough without having to take onboard further linguistic and cultural differences that might affect how language disorders manifest and are treated.

2 In a Response to Intervention framework, three tiers of intervention/prevention are identified, with tier 3 referring to the level of individualized intervention needed for children with the most severe problems.

## References

[jlcd12335-bib-0001] American Speech‐Language‐Hearing Association , 2016, Eligibility and dismissal in schools [online] (available at: http://www.asha.org/slp/schools/prof-consult/eligibility0 (accessed on 29 May 2017).

[jlcd12335-bib-0002] Bishop, D. V. M. , 1994, Is specific language impairment a valid diagnostic category? Genetic and psycholinguistic evidence. Philosophical Transactions of the Royal Society, Series B, 346, 105–111. 10.1098/rstb.1994.0134 7886145

[jlcd12335-bib-1002] Bishop, D. V. M. , 2010, Which neurodevelopmental disorders get researched and why?. PLOS One, 5, e15112. 21152085 10.1371/journal.pone.0015112 PMC2994844

[jlcd12335-bib-0003] Bishop, D. V. M. , 2014, Ten questions about terminology for children with unexplained language problems. International Journal of Language and Communication Disorders, 49, 381–415. 25142090 10.1111/1460-6984.12101 PMC4314704

[jlcd12335-bib-1003] Bishop, D. V. M. , Clark, B. , Conti‐Ramsden, G. , Norbury, C. F. and Snowling, M. J. , 2012, RALLI: An internet campaign for raising awareness of language learning impairments. Child Language Teaching & Therapy, 28, 259–262.

[jlcd12335-bib-1004] Bishop, D. V. M. and Edmundson, A. , 1987, Language‐impaired four‐year‐olds: distinguishing transient from persistent impairment. Journal of Speech and Hearing Disorders, 52, 156–173. 3573746 10.1044/jshd.5202.156

[jlcd12335-bib-0004] Bishop, D. V. M. , Snowling, M. J. , Thompson, P. A. , Greenhalgh, T. and the catalise consortium , 2016, CATALISE: a multinational and multidisciplinary Delphi consensus study. Identifying language impairments in children. PLOS One, 11, e0158753. 27392128 10.1371/journal.pone.0158753 PMC4938414

[jlcd12335-bib-0005] Bishop, D. V. M. , Snowling, M. J. , Thompson, P. A. , Greenhalgh, T. and the catalise consortium , 2017, CATALISE: a multinational and multidisciplinary Delphi consensus study of problems with language development. Phase 2. Terminology. Journal of Child Psychology and Psychiatry, 58, 1068–1080. 28369935 10.1111/jcpp.12721 PMC5638113

[jlcd12335-bib-0006] Brosnan, M. and Mills, E. , 2016, The effect of diagnostic labels on the affective responses of college students towards peers with ‘Asperger's Syndrome’ and ‘Autism Spectrum Disorder’. Autism, 20, 388–394. 26045542 10.1177/1362361315586721

[jlcd12335-bib-0007] Cole, K. N. , Dale, P. S. and Mills, P. E. , 1990, Defining language delay in young children by cognitive referencing: are we saying more than we know? Applied Psycholinguistics, 11, 291–302.

[jlcd12335-bib-0008] Crabtree, J. W. , Haslam, S. A. , Postmes, T. and Haslam, C. , 2010, Mental health support groups, stigma, and self‐esteem: positive and negative implications of group identification. Journal of Social Issues, 66, 553–569.

[jlcd12335-bib-0009] Dollaghan, C. A. , 2011, Taxometric analyses of specific language impairment in 6‐year‐old children. Journal of Speech, Language and Hearing Research, 54, 1361–1371. 10.1044/1092-4388(2011/10-0187) 21646422

[jlcd12335-bib-0010] Ebbels, S. , 2014, Introducing the SLI debate. International Journal of Language and Communication Disorders, 49, 377–380. 25142089 10.1111/1460-6984.12119 PMC4312950

[jlcd12335-bib-0011] Elliott, J. G. and Grigorenko, E. L. , 2014, The Dyslexia Debate (Cambridge: Cambridge University Press).

[jlcd12335-bib-0012] Harris, J. C. , 2013, New terminology for mental retardation in DSM‐5 and ICD‐11. Current Opinion in Psychiatry, 26, 260–262. 23507999 10.1097/YCO.0b013e32835fd6fb

[jlcd12335-bib-0013] Hasson, F. , Keeney, S. and Mckenna, H. , 2000, Research guidelines for the Delphi survey technique. Journal of Advanced Nursing, 32, 1008–1015. 11095242

[jlcd12335-bib-0014] Hawker, K. , Ramirez‐Inscoe, J. , Bishop, D. V. M. , Twomey, T. , O'Donoghue, G. M. and Moore, D. R. , 2008, Disproportionate language impairment in children using cochlear implants. Ear and Hearing, 29, 467–471. 18453886 10.1097/AUD.0b013e318167b857

[jlcd12335-bib-0015] International Dyslexia Association , 2012, International Dyslexia Association requests reinstatement of the term ‘dyslexia’ in American Psychiatric Association's DSM‐5 [online] (available at: http://www.prnewswire.com/news-releases/international-dyslexia-association-requests-reinstatement-of-the-term-dyslexia-in-american-psychiatric-associations-dsm-5-157488625.html) (accessed on 29 May 2017).

[jlcd12335-bib-0016] Leonard, L. B. , 1991, Specific language impairment as a clinical category. Language, Speech, and Hearing Services in Schools, 22, 66–68. 10.1044/0161-1461(2009/08-0096) PMC2897931 20421612

[jlcd12335-bib-0017] Macharey, G. and Von Suchodoletz, W. , 2008, Perceived stigmatization of children with speech–language impairment and their parents. Folia Phoniatrica et Logopaedica, 60, 256–263. 18765946 10.1159/000151763

[jlcd12335-bib-0018] Mcrae, J. F. , Clayton, S. , Fitzgerald, T. W. , Kaplanis, J. , Prigmore, E. , Rajan, D. *et al*, 2017, Prevalence and architecture of de novo mutations in developmental disorders. Nature, 542, 433. 28135719 10.1038/nature21062 PMC6016744

[jlcd12335-bib-0019] Reilly, S. , Bishop, D. V. M. and Tomblin, B. , 2014a, Terminological debate over language impairment in children: forward movement and sticking points. International Journal of Language and Communication Disorders, 49, 452–462. 25142092 10.1111/1460-6984.12111 PMC4312775

[jlcd12335-bib-0020] Reilly, S. , Tomblin, B. , Law, J. , Mckean, C. , Mensah, F. , Morgan, A. , Goldfeld, S. , Nicholson, J. and Wake, M. , 2014b, SLI: a convenient label for whom? International Journal of Language and Communication Disorders, 49, 416–451. 25142091 10.1111/jlcd.1460-6984.12102 PMC4303922

[jlcd12335-bib-0021] Sonuga‐Barke, E. J. S. , 1998, Categorical models of childhood disorder: a conceptual and empirical analysis. Journal of Child Psychology and Psychiatry, 39, 115–133. 9534089

[jlcd12335-bib-0022] Taylor, L. M. , Hume, I. R. and Welsh, N. , 2010, Labelling and self‐esteem: the impact of using specific vs. generic labels. Educational Psychology, 30, 191–202.

